# Horizontal Transmission of "*Candidatus *Liberibacter solanacearum" by *Bactericera cockerelli* (Hemiptera: Triozidae) on *Convolvulus* and *Ipomoea* (Solanales: Convolvulaceae)

**DOI:** 10.1371/journal.pone.0142734

**Published:** 2015-11-10

**Authors:** Glenda L. Torres, W. Rodney Cooper, David R. Horton, Kylie D. Swisher, Stephen F. Garczynski, Joseph E. Munyaneza, Nina M. Barcenas

**Affiliations:** 1 Heritage University, Department of Natural Sciences, Toppenish, WA, 98948, United States of America; 2 USDA/Agricultural Research Service, Yakima Agricultural Research Laboratory, Wapato, WA, 98951, United States of America; University of Idaho, UNITED STATES

## Abstract

“*Candidatus* Liberibacter solanacearum” (Proteobacteria) is an important pathogen of solanaceous crops (Solanales: Solanaceae) in North America and New Zealand, and is the putative causal agent of zebra chip disease of potato. This phloem-limited pathogen is transmitted to potato and other solanaceous plants by the potato psyllid, *Bactericera cockerelli* (Hemiptera: Triozidae). While some plants in the Convolvulaceae (Solanales) are also known hosts for *B*. *cockerelli*, previous efforts to detect Liberibacter in Convolvulaceae have been unsuccessful. Moreover, studies to determine whether Liberibacter can be acquired from these plants by *B*. *cockerelli* are lacking. The goal of this study was to determine whether horizontal transmission of Liberibacter occurs among potato psyllids on two species of Convolvulaceae, sweet potato (*Ipomoea batatas*) and field bindweed (*Convolvulus arvensis*), which grows abundantly in potato growing regions of the United States. Results indicated that uninfected psyllids acquired Liberibacter from both *I*. *batatas* and *C*. *arvensis* if infected psyllids were present on plants concurrently with the uninfected psyllids. Uninfected psyllids did not acquire Liberibacter from plants if the infected psyllids were removed from the plants before the uninfected psyllids were allowed access. In contrast with previous reports, PCR did detect the presence of Liberibacter DNA in some plants. However, visible amplicons were faint and did not correspond with acquisition of the pathogen by uninfected psyllids. None of the plants exhibited disease symptoms. Results indicate that horizontal transmission of Liberibacter among potato psyllids can occur on Convolvulaceae, and that the association between Liberibacter and Convolvulaceae merits additional attention.

## Introduction

"*Candidatus* Liberibacter solanacearum" (also known as "*Ca*. Liberibacter psyllaurous") is an important pathogen of solanaceous crops (Solanales: Solanaceae), and is the putative causal agent of zebra chip disease of potato [1]. Liberibacter is phloem-limited and is transmitted to plants by the potato psyllid, *Bactericera cockerelli* (Hemiptera: Triozidae) [2]. This pathogen is managed in potatoes primarily using calendar-based insecticide applications targeting the psyllid vector [1]. The use of calendar-based treatments rather than targeted applications is needed because of insufficient knowledge regarding the timing and sources of infected psyllids entering crop fields [3]. Current research efforts seek to improve predictions on the timing and sources of Liberibacter-infected psyllids migrating to crops by identifying weed hosts that support populations of these psyllids.

Although *B*. *cockerelli* prefers Solanaceae as hosts, this psyllid can reproduce and develop on some plants in the Convolvulaceae (Order: Solanales) including sweet potato (*Ipomoea batatas*) and the perennial weed, field bindweed (*Convolvulus arvensis*) [4,5,6] (Horton unpublished). *C*. *arvensis* is abundant in potato growing regions of the United States, presenting the need to determine whether this plant is important for Liberibacter epidemiology. Previous studies suggested that neither *I*. *batatas* [7] nor *C*. *arvensis* [8] are hosts for Liberibacter, and that *I*. *batatas* or *C*. *arvensis* plants exposed to the pathogen do not develop visible disease symptoms. Because PCR has failed to detect Liberibacter in *I*. *batatas* or *C*. *arvensis*, previous investigators assumed that *B*. *cockerelli* could not acquire Liberibacter from these plants [7,9]. Although the failure to detect Liberibacter from *I*. *batatas* and *C*. *arvensis* suggests that psyllids cannot inoculate these plants with Liberibacter, the assumption that they cannot acquire the pathogen from these plants has not been specifically tested. Acquisition of Liberibacter by *B*. *cockerelli* on potato does not always correspond with pathogen detection, but does correspond with predicted dispersal patterns of the pathogen from inoculation leaves [10]. It therefore seems possible that horizontal transmission of Liberibacter can occur on plants without plant infection. Still, the assumption that *B*. *cockerelli* is unable to acquire Liberibacter from *I*. *batatas* was the basis for the experimental design and interpretation of results in a previous study [9]. To better understand the potential role of *C*. *arvensis* in Liberibacter epidemiology and to better utilize *I*. *batatas* in future studies on psyllid-Liberibacter interactions, the primary goal of our study was to determine whether *B*. *cockerelli* can acquire Liberibacter from two species of Convolvulaceae, *I*. *batatas* and *C*. *arvensis*.

## Materials and Methods

### Plants and Insects

Liberibacter-infected and uninfected psyllids were obtained from separate laboratory colonies established using psyllids of the Western haplotype [11]. Infected psyllids harbored Liberibacter-haplotype B based on the haplotyping methods described by Wen et al. [12]. Both colonies were maintained on potato ‘Ranger Russet’ and tomato ‘Moneymaker’ at 25°C with a 16:8 (L:D) hour photoperiod and 50% relative humidity. Adult psyllids from both colonies were periodically tested for the presence or absence of Liberibacter using PCR (see PCR Diagnosis of Liberibacter). Prior to experiments, insect sex was determined under 25× magnification using an Olympus SZX7 microscope (Olympus America Inc., Central Valley, PA.).

*I*. *batatas* plants were grown from fresh-market tubers in 900-cm³ plastic pots with a 1:3 ratio of sand and Miracle-Gro (Scotts Miracle-Gro Company). *C*. *arvensis* plants were grown from seed in 900-cm³ plastic pots with a 1:2 ratio of sand and Sta-Green All Purpose Potting Mix Plus Fertilizer (Waupaca Northwoods, LLC, Waupaca, WI). The seeds were collected from field populations of *C*. *arvensis* growing near Selah, WA (46° 40’ 57.325” N 120° 31’ 43.938” W); no permit was required for collection of seeds. All plants were grown in a greenhouse with supplemental lighting to provide a 16:8 (L:D) hour photoperiod.

### Acquisition of Liberibacter by Psyllids on Plants Co-Infested with Infected Psyllids

**Fig 1:**
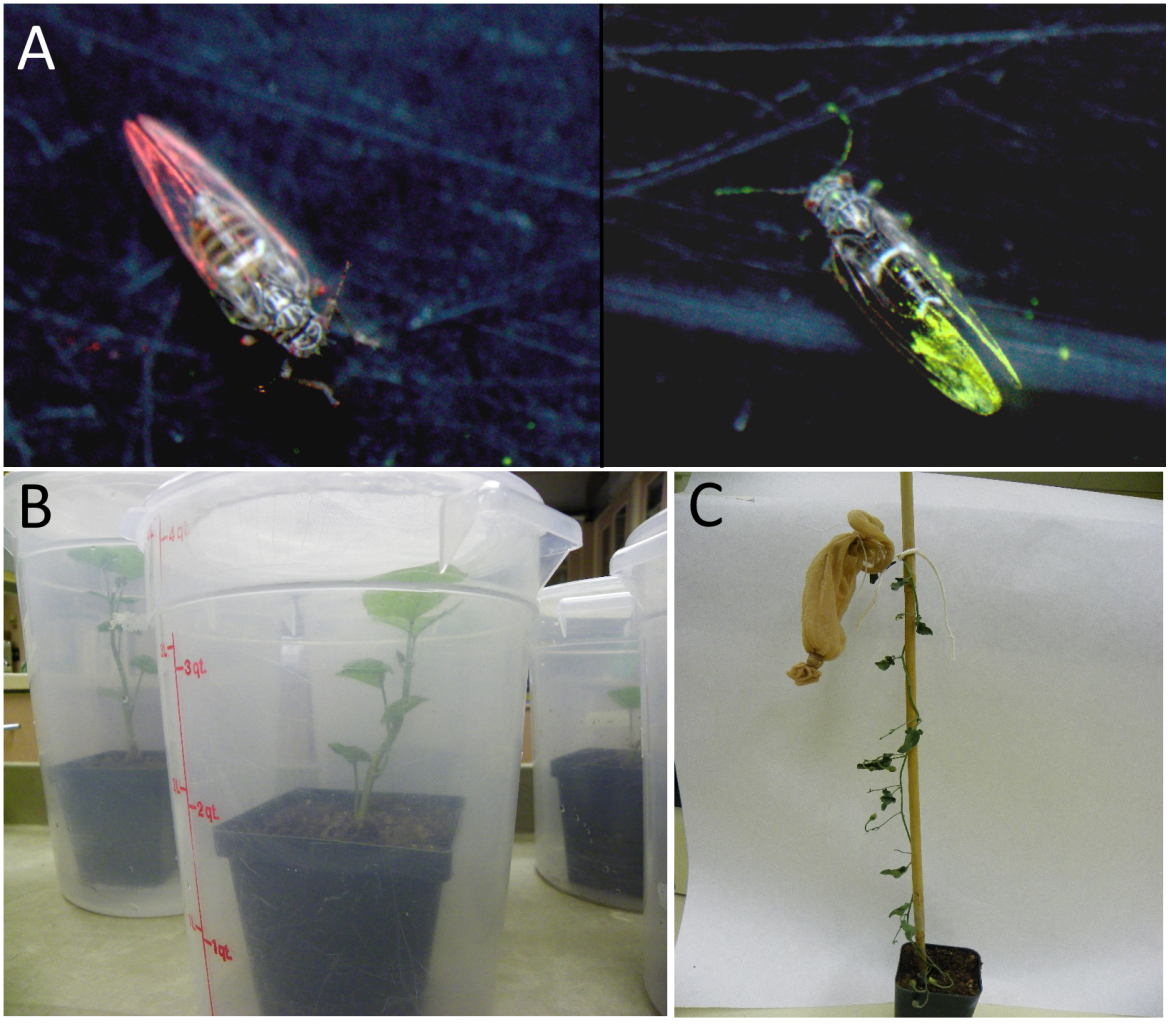
Acquisition of Liberibacter by uninfected psyllids on plants co-infested with infected psyllids. Wings of Liberibacter-infected and uninfected psyllids were lightly marked with fluorescent powder (A) before confining them concurrently to whole *I*. *batatas* plants (B) or to the terminals (4–5 leaves) of *C*. *arvensis* plants (C).

The wings of infected and uninfected psyllids were lightly marked with different colors of fluorescent powder (BioQuip Products, Rancho Dominguez, CA) to allow us to distinguish initial infection status at the completion of the assay (Fig 1A). Five Liberibacter-infected and five uninfected psyllids were confined to a whole *I*. *batatas* plant (Fig 1B) or the terminal (~5 developing leaves) of a *C*. *arvensis* plant (Fig 1C). Plants infested with 10 uninfected psyllids were used as controls. Because another Liberibacter species, "*Ca*. Liberibacter asiaticus," is sexually transmitted among Asian citrus psyllids, *Diaphornia citri* [13], each plant received only psyllids of one sex. About equal numbers of plants received male or female psyllids. Infested plants were maintained in a growth chamber (model I30BLL, Percival Scientific Inc., Perry IA) kept at 25°C with a 16:8 (L:D) hour photoperiod for one week. Psyllids were then collected, separated by initial infection status (as known by the color of fluorescent powder; Fig 1A), and assayed for presence of Liberibacter using PCR (see PCR Diagnosis of Liberibacter). Three trials were conducted with *I*. *batatas* and two trials with *C*. *arvensis*. Each trial included different cohorts of plants and insects (5–10 replications per trial; see Table 1A for total sample sizes), and two control plants.

**Table 1:** Acquisition of Liberibacter by psyllids on *I*. *batatas* and *C*. *arvensis*.

Experimental Objective	Plant	Infected groups of psyllids^a^	Acquisition rate ^b^
A) Plants Co-Infested with Uninfected and Infected Psyllids	*I*. *batatas*	15/15	9/15
	*C*. *arvensis*	19/19	2/19
B) Plants Previously Exposed to Infected Psyllids	*I*. *batatas*, 48-h acquisition access period ^c^	5/5	0/5
	*I*. *batatas* 1-week acquisition access period ^d^	12/12	0/12
	*C*. *arvensis* 1-week acquisition access period ^d^	10/12	0/10

^a^ PCR confirmation that psyllids obtained from the infected colonies carried Liberibacter; number of infected groups of insects divided by the total number of experimental plants

^b^ Number of plants on which uninfected psyllids acquired Liberibacter divided by the total number of plants exposed to Liberibacter. PCR amplicons were observed for positive controls, but not for negative or single primer controls. None of the psyllids from control plants were infected with Liberibacter.

^c^ Uninfected psyllids placed on plants for 48 hours immediately after an inoculation access period of 48 hours.

^d^ Uninfected psyllids placed on plants for 5 days, three weeks after initially releasing infected psyllids on plants for a 1-week inoculation access period.

### Acquisition of Liberibacter from Plants Previously Exposed to Infected Psyllids

Five Liberibacter-infected adult male psyllids were confined to single leaves on each of five *I*. *batatas* plants using a sleeve cage. Male psyllids were used to inoculate plants to avoid infestations of plants with offspring. The infected psyllids were removed from plants after 48 hours, and then replaced with five uninfected psyllids. Two plants previously exposed to uninfected psyllids were used as controls. The initially uninfected psyllids and remaining leaves were collected after 48 hours and tested for the presence of Liberibacter using PCR.

In a separate study, five infected adult male psyllids were confined to a single leaf using a sleeve cage on each *I*. *batatas* or *C*. *arvensis* plant for a 1-week inoculation access period. Psyllids were removed after 1 week and tested with PCR to confirm infection status. Three weeks after initial releases of infected psyllids, five uninfected psyllids were confined to the plants for five days and then collected to determine whether they had acquired Liberibacter. The terminals of each plant were also collected and assayed for the presence of Liberibacter. This experiment was conducted twice (trial) using *I*. *batatas* plants and three times using *C*. *arvensis* plants. Each trial included 4–6 replications (see Table 1B for total sample sizes) and two control plants.

### Dispersal of Liberibacter among Leaves

**Fig 2:**
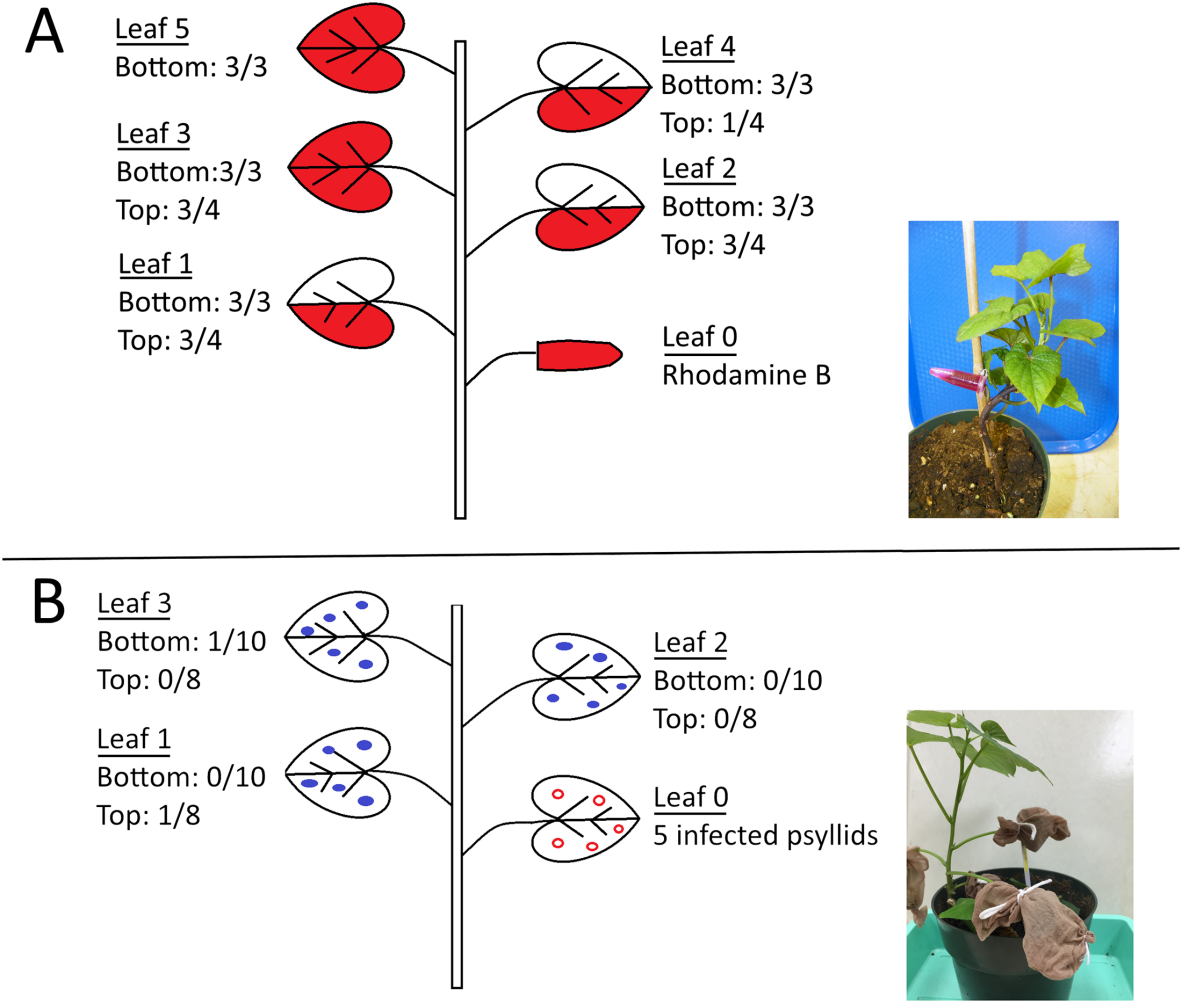
Within-plant dispersal of Liberibacter. Rhodamine B introduced into leaf position 0 migrated (shaded area) from each of leaves 1 through 5 following orthostichous patterns (A). Uninfected psyllids (closed circles) acquired Liberibacter that migrated from inoculation leaves (leaf 0) infested with infected psyllids (open circles) (B). Inset photographs show the experimental setups. Experiments were conducted from both the bottom of the plant ("bottom"; second fully-expanded leaf from the soil designated as leaf 0) and the top of the plant ("top"; fourth fully expanded leaf from the terminal leaves designated as leaf 0). Numbers indicate the number of leaves accumulating dye (A) divided by the total number of sample leaves (A), or leaves on which psyllids acquired Liberibacter divided by the total number of sample leaves (B).

Vascular connectivity of leaves of *I*. *batatas* was examined by observing the migration of rhodamine B (Matheson Coleman and Bell, Cincinnati, OH) from an inoculation leaf to other leaves using the methods described by Cooper et al. [10]. An inoculation leaf designated as leaf 0 was excised, and the cut petiole attached to the plant was inserted into a 1.5-ml microfuge tube filled with a solution of 0.25% rhodamine B in 30 mM EDTA (Fig 2A inset). After 6 hours at 25°C, the presence or absence of dye was observed in the leaves above leaf 0. The experiment was conducted twice (trial). In the first trial, the second leaf up from the soil was designated as leaf 0 and the five leaves above this leaf (designated as leaves 1 through 5) were observed for accumulation of dye. In the second trial, the fourth fully expanded leaf down from the terminal was designated as leaf 0 and the four leaves above this leaf were observed for dye accumulation.

After mapping the route of rhodamine B among leaves of *I*. *batatas*, we determined whether Liberibacter disperses from initial sites of inoculation and is acquired by uninfected psyllids on leaves that share a vascular connection with the inoculated leaf. Using sleeve cages (Fig 2B inset), five infected psyllids were confined to inoculation leaves (leaf 0) and five uninfected psyllids were confined to each of three leaves above the inoculation leaf (leaves 1 through 3). The plants were placed in a greenhouse with supplemental lighting providing a 16:8 (L:D) hours photoperiod, and psyllids were collected after 1 week to determine whether they acquired the pathogen. In two separate trials, each with five plants, the second-fully expanded leaf up from the soil was designated as leaf 0. In a third trial with eight plants, the fourth leaf down from the plant terminal was designated as leaf 0. All three trials included two control plants each with ten uninfected psyllids.

### Acquisition of Liberibacter without a Host

The purpose of this experiment was to confirm that Liberibacter cannot be horizontally transmitted among psyllids of the same sex in the absence of a host plant (e.g. through honeydew contamination). Five Liberibacter-infected and uninfected adult psyllids of the same sex were confined to a petri dish. Moist filter paper was placed at the bottom of the petri dish and each dish was sealed with parafilm to prevent the desiccation of the psyllids. The wings of each insect were lightly marked with fluorescent powder to differentiate between infected and uninfected psyllids. The petri dishes were placed in a growth chamber (model I30BLL, Percival Scientific Inc., Perry IA) maintained at 25°C with a 16:8 (L:D) h photoperiod. After 48 h, the infected psyllids were collected to confirm that they harbored Liberibacter. Duration of 48 h was chosen because preliminary studies indicated substantial mortality of psyllids held without a host plant for longer than 48 h. Each group of 5 initially uninfected psyllids were transferred to a potato plant and confined to a single leaf using a sleeve cage. After a 1-week incubation period, the uninfected psyllids were collected to determine if they acquired Liberibacter.

### PCR Diagnosis of Liberibacter

DNA was extracted from plants and psyllids using the cetyltrimethylammonium bromide precipitation method [14,15]. DNA was extracted from insects pooled from each group of five infected or previously uninfected psyllids from each plant, and was suspended in 50 μl of water. DNA from groups of 10 psyllids from control plants and DNA from plant samples were suspended in 100 μl of water.

The presence or absence of Liberibacter in psyllids was assessed by amplifying a region of an outer membrane protein gene using the primers OMBf- GGC GTG GTT ATA AGC AGA G and OMBr-ATC TAC ACG CGG ACC TAT AC [15]. Each 10 μl reaction contained Invitrogen Amplitaq Gold 360 PCR Master Mix (Invitrogen, Carlsbad, CA), 500 nM of each primer, and 1 μl of DNA template. The PCR conditions were 95°C for 2 min, then 40 cycles of 95°C for 20 s, 65°C for 10 s, and 72°C for 70 s, followed by a final incubation at 72°C for 15 min [15]. Positive controls, negative controls, and single-primer controls were included for PCRs. The presence or absence of 605-bp PCR amplicons was observed on 1% agarose gels stained with ethidium bromide. Crosslin et al [15] reported that this PCR procedure is sensitive enough to detect a single Liberibacter-infected psyllid in pooled samples of as many 10 psyllids.

Because detection of Liberibacter in plants can be difficult [16,17], all plants were assayed for Liberibacter using the primer set for outer membrane protein and a second primer set for 16S using primers OA2- GCG CTT ATT TTT AAT AGG AGC GGC A and OI2c-GCC TCG CGA CTT CGC AAC CCA T [18]. PCR conditions using primers OA2/OI2c were 94°C for 5 min, then 35 cycles of 94°C for 30 s, 66°C for 30 s, and 72°C for 60 s, followed by a final incubation at 72°C for 10 min [18]. The presence or absence of 1168-bp amplicons were observed on a 1% agarose gel stained with ethidium bromide. Observed bands were excised from gels and purified using GenElute minus EtBr spin columns (Sigma, St. Louis, MO), and were cloned using a TOPO TA cloning kit with TOP10 *E*. *coli* chemically competent cells (Invitrogen, Carlsbad, CA). Plasmid DNA was extracted from selected colonies using the QIAprep spin mini prep kit (Qiagen, Valencia, CA), and DNA clones were sequenced by MC Laboratories (MC Lab, San Francisco, CA). Sequences were analyzed using BLASTn [19] function on the NCBI website (http://www.ncbi.nlm.nih.gov/).

A third primer set, SSR-1F- TTA TTT TGA GAT GGT TTG TTA AAT G and SSR-1R-TAT TAT CAT TCT ATT GCC TAT TTC G [20,12] was used to assay plants for Liberibacter if the first two primer sets (OMBf/OMBr and OA2/OI2c) produced conflicting results. Each 10 μl reaction contained Advantage II Taq polymerase and 1× PCR buffer (Clonetech Laboratories, Inc., Mountain View, CA), 200 nM dNTPS, 125 nM of each primer, and 1 μl of sample DNA. The PCR conditions were 94°C for 5 min, then 40 cycles of 94°C for 10 s, 58°C for 10 s, and 72°C for 15 s, followed by a final incubation at 72°C for 5 min (Swisher unpublished). A 1.5% agarose gel stained with ethidium bromide was used to observe the presence or absence of 180-bp products corresponding with the presence of Liberibacter haplotype B.

## Results and Discussion

### Detection of Liberibacter in Psyllids

**Fig 3:**
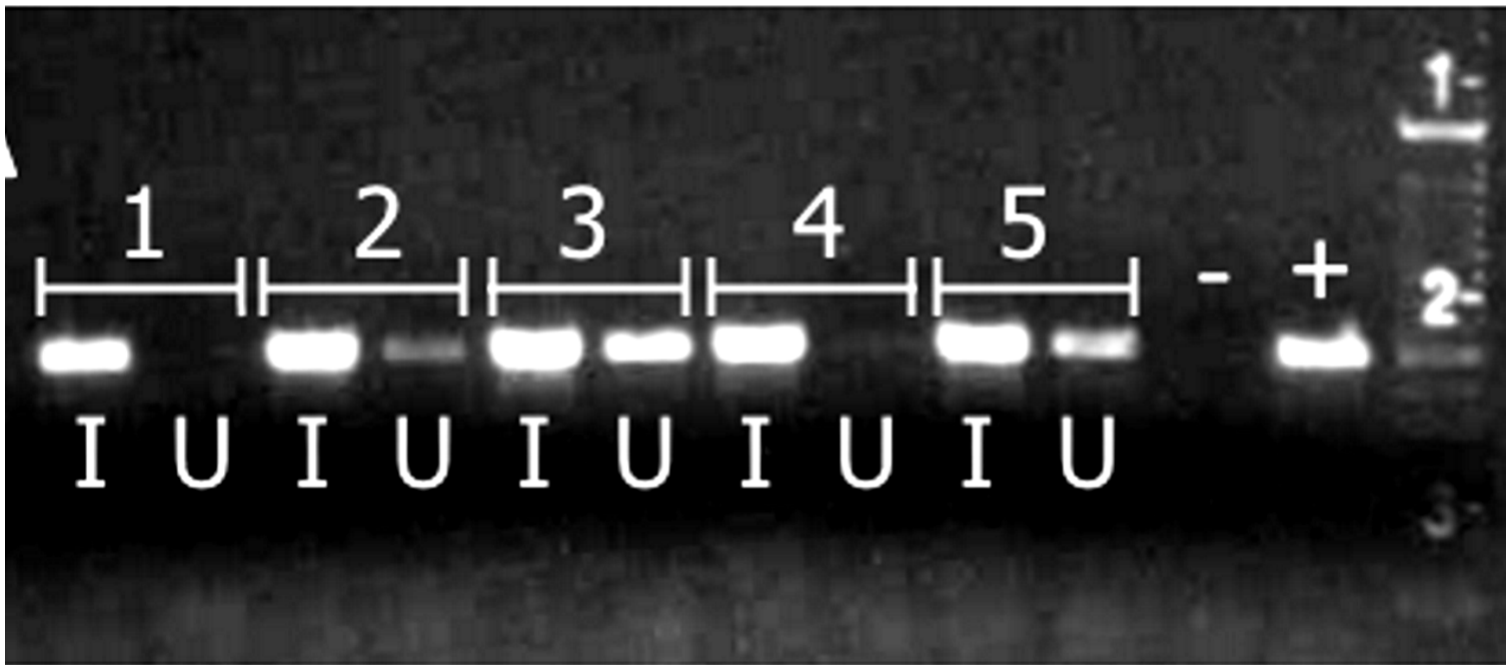
Sample gel of Liberibacter detection in *B*. *cockerelli*. PCR was performed on each group of five infected psyllids (I) and five initially uninfected psyllids (U) that were simultaneously confined to each *I*. *batatas* plant for 1 week. Individual plants are represented by numbers. In this sample gel, initially uninfected psyllids on plants 2, 3, and 5 acquired Liberibacter, but psyllids on plants 1 and 4 did not.

When concurrently confined to plants with Liberibacter-infected psyllids, the initially uninfected psyllids acquired the pathogen on 60% of *I*. *batatas* plants and on 11% of *C*. *arvensis* plants (Table 1A; Fig 3). However, none of the uninfected psyllids acquired Liberibacter from plants if infected psyllids were removed from the plants before acquisition access (Table 1B). This was observed regardless of whether uninfected psyllids were exposed to the inoculated plants immediately following a 48-hour inoculation access period, or exposed to plants 2 weeks after a 1-week inoculation access period (Table 1B). Psyllids did not acquire the pathogen when confined within petri dishes with infected psyllids but without plants, demonstrating that the plants were involved in horizontal transmission of Liberibacter. These results indicate that psyllids were capable of acquiring Liberibacter from *I*. *batatas* and *C*. *arvensis*, but only if Liberibacter-infected psyllids were simultaneously present on plants with the uninfected psyllids.

To further explicate the horizontal transmission of Liberibacter among psyllids concurrently infesting plants in the Convolvulaceae, we tested the ability for uninfected psyllids to acquire Liberibacter from *I*. *batatas* leaves that shared vascular connections with leaves harboring infected psyllids. Vascular architecture of *I*. *batatas* was determined by observing the migration of rhodamine B from inoculation leaves. Unlike previous observations on potato [10], the dye accumulated in all leaves above the inoculation leaf thus demonstrating at least partial vascular connections among these leaves (Fig 2A). Accumulation of the dye followed orthostichous patterns wherein the dye tended to accumulate in half of each leaf not vertically aligned with the inoculation leaf (leaves 1, 2, and 4), and in the whole of each leaf vertically aligned with the inoculation leaf (leaves 3 and 5). These migration patterns were observed in each trial, regardless of the position of the inoculation leaf. Since experiments with rhodamine B indicated at least partial vascular connectivity between inoculation leaves and all upper leaves, we confined infected psyllids on an inoculation leaf and uninfected psyllids on each of the three leaves above this leaf. Results demonstrated that Liberibacter dispersed among leaves of *I*. *batatas* and was acquired within 1 week by uninfected psyllids feeding on leaves distant from the inoculation leaf (Fig 2B). These results were consistent with a previous study in which psyllids acquired Liberibacter from potato leaves distant from the inoculation leaf within the same access period [10].

### Detection of Liberibacter in Plant Samples

**Fig 4:**
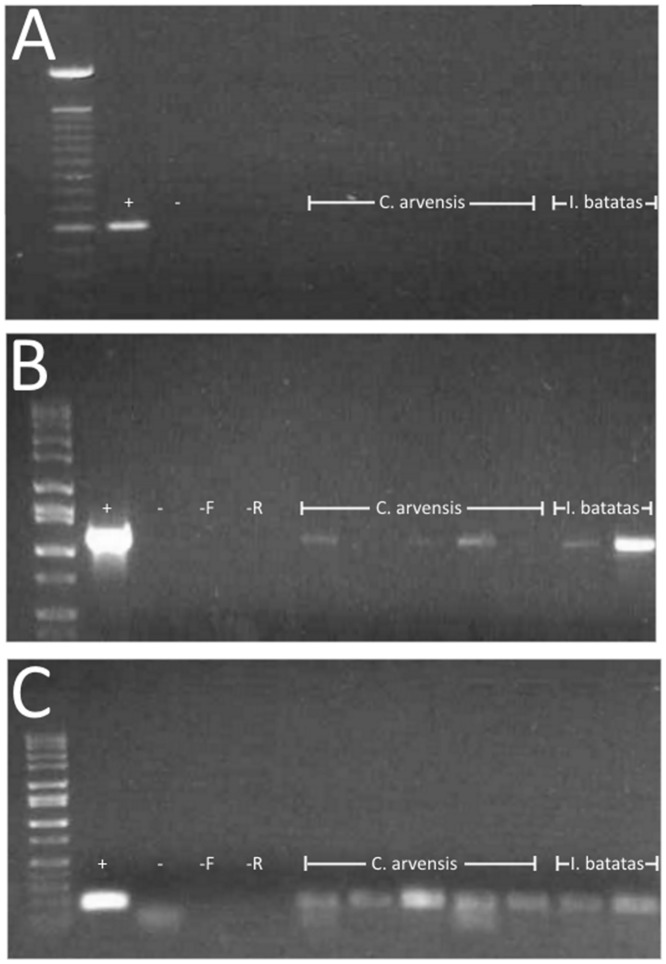
Sample gel of Liberibacter detection in *I*. *batatas* and *C*. *arvensis*. PCR was performed on all plant samples using primer set OMBf/OMBr producing a 605-bp product (A), and using primer set OA2/OI2c producing a 1168-bp product (B). Primer set SSR-1F/SSR-1R, which produced a 180-bp product for Liberibacter haplotype B (C), was performed on samples from which conflicting results were obtained with the first two primer sets.

Liberibacter was not detected in *I*. *batatas* or *C*. *arvensis* 2-days post inoculation. However, PCR using two different primer sets produced conflicting results on the presence of Liberibacter in plants 3-weeks post inoculation. Although PCR using the primer set for outer membrane protein (OMBf/OMBr) did not detect the presence of the pathogen in any of the plants (Fig 4A), faint bands were observed in 2 of 12 *I*. *batatas* (SP) plants and in 5 of 10 *C*. *arvensis* (BW) plants when PCR was performed using primers for 16S (OA2/OI2c) (Fig 4B). The sequences of these 16S products (archived as GenBank accession numbers KT354969-KT354975) were 99% identical (1109/1111 base pairs) with those of "*Ca*. Liberibacter solanacearum" (for example GenBank accession number KF776422). Reevaluating these samples using SSR primers [20] and high fidelity PCR reagents confirmed the presence of Liberibacter DNA (Fig 4C). Our observation that Liberibacter DNA can be detected in *I*. *batatas* conflicts with previous reports [7,8]. These conflicting results may be due to differences in plant varieties, PCR methods, or environmental conditions in which plants were maintained. Regardless of the detection of Liberibacter DNA none of the plants exhibited disease symptoms, which was consistent with the previous reports, and psyllids did not acquire the pathogen from any of the plants from which Liberibacter DNA was detected.

The detection of Liberibacter DNA in *I*. *batatas* and *C*. *arvensis* and the lack of corresponding acquisition by *B*. *cockerelli* indicate that our understanding of the interactions between Liberibacter and plants in the Convolvulaceae is incomplete. It is possible that these plants are asymptomatic hosts in which the titers of the pathogen are too low to be acquired by psyllids or consistently detected using conventional PCR. If plants in the Convolvulaceae do indeed support Liberibacter, the pathogen was apparently unavailable for acquisition by uninfected psyllids after infected psyllids were removed from the plants (Table 1). Genes involved in cell-adhesion have been identified from "*Ca*. Liberibacter solanacearum" [21] so binding to the phloem walls or sieve plates seems possible, and would likely prevent psyllids from acquiring the pathogen. Alternatively, other factors required for long-term survival and reproduction of Liberibacter may not be present in these Convolvulaceae.

### Possible Mechanisms of Horizontal Transmission

Our observation that psyllids did not acquire Liberibacter after infected psyllids were removed from plants (Table 1) suggests that transmission may have occurred while the pathogen passively migrated through the phloem and before it became unavailable or died due to poor or non-host factors. This interpretation is consistent with a previous study in which acquisition of Liberibacter on potato did not always correspond with detection of the pathogen in the leaves, but did correspond with predicted dispersal patterns of Liberibacter from inoculation leaves [10]. Although horizontal transmission of arboviruses occurs among ticks on nonviremic animal hosts [22], this form of transmission among vectors has not been observed for plant pathogens. Horizontal transmission via passive migration through phloem, if occurring, could have implications beyond Liberibacter epidemiology. Since all known phloem-limited bacteria rely upon Hemipteran insect vectors, Bové and Garnier [23] proposed that phloem-limited plant pathogens evolved from insect endosymbionts which accidentally colonized the salivary glands of their vectors. Horizontal transmission by way of passive movement through plant phloem could provide a mechanism for evolution of Hemipteran-vectored and phloem-limited plant pathogens by providing a selective advantage to endosymbionts colonizing insect salivary glands. Our hypothesis that horizontal transmission of bacteria occurs among insects via passive migration through plant vascular highways is intriguing and potentially important, but requires further research involving different insect-plant-bacteria associations before this hypothesis can be unequivocally accepted.

## Conclusions

Results of this study indicate that "*Ca*. Liberibacter solanacearum" is transmitted from infected to uninfected potato psyllids on *I*. *batatas* and *C*. *arvensis*, two plants not previously known to support the pathogen. However, transmission occurred only when uninfected and infected psyllids were present on the plants simultaneously, and detection of Liberibacter in Convolvulaceae using conventional PCR did not correspond with acquisition by psyllids. Our findings indicate that our understanding of the interactions between Liberibacter and plants in the Convolvulaceae is incomplete, and demonstrate the need for further study using more sophisticated techniques including quantitative PCR to quantify Liberibacter levels in *I*. *batatas* and *C*. *arvensis*. Our findings may help researchers develop landscape-level management strategies for Liberibacter if the ecological implications of Liberibacter transmission on Convolvulaceae were better understood. *C*. *arvensis* is an invasive weed common in potato and tomato growing regions of the United States which serves as a host for *B*. *cockerelli* before and after crops are present. Although our results show that transmission of Liberibacter among psyllids occurs on this weed, comprehensive field surveys, and studies to determine rates of acquisition on *C*. *arvensis* are needed to determine whether this weed is important for Liberibacter epidemiology. Finally, studies are currently underway to determine whether interspecific transmission between *B*. *cockerelli* and *B*. *maculipennis*, a native psyllid commonly found on *C*. *arvensis*, occurs on plants in the Convolvulaceae.
